# Respiratory Viruses in Wastewater Compared with Clinical Samples, Leuven, Belgium

**DOI:** 10.3201/eid3001.231011

**Published:** 2024-01

**Authors:** Annabel Rector, Mandy Bloemen, Marijn Thijssen, Bram Pussig, Kurt Beuselinck, Marc Van Ranst, Elke Wollants

**Affiliations:** KU Leuven Rega Institute, Louvain, Belgium (A. Rector, M. Bloemen, M. Thijssen, M. Van Ranst, E. Wollants);; KU Leuven Academic Center for General Practice, Louvain (B. Pussig);; University Hospitals Leuven, Louvain (K. Beuselinck, M. Van Ranst).

**Keywords:** respiratory infections, viruses, wastewater-based epidemiology, acute respiratory tract infection, respiratory panel, influenza, RSV, enterovirus, rhinovirus, EV-D68, coronavirus respiratory syncytial virus, enterovirus D68, Belgium

## Abstract

In a 2-year study in Leuven, Belgium, we investigated the use of wastewater sampling to assess community spread of respiratory viruses. Comparison with the number of positive clinical samples demonstrated that wastewater data reflected circulation levels of typical seasonal respiratory viruses, such as influenza, respiratory syncytial virus, and enterovirus D68.

Since the COVID-19 pandemic began, wastewater-based surveillance has been used to track circulation levels of SARS-CoV-2 (*1*,*2*). For that purpose, we began collecting samples from a regional wastewater treatment plant in Leuven, Belgium, in December 2020. We found wastewater-based surveillance was an objective indicator of SARS-CoV-2 community circulation, which can be highly valuable when testing is limited (*3*).

Many persons with acute respiratory infections (ARI) do not seek medical care, thereby enabling those infections to go undetected. Obtaining detailed information on the circulation of respiratory viruses in the community is key to elucidating their societal burden. This knowledge could enable better prediction and management of major outbreaks and could guide physicians in diagnosis. The current approach, usually based on limited reporting by sentinel physicians and laboratories, can lead to substantial data bias. We explored whether wastewater sampling can provide an alternative method for monitoring circulation of respiratory pathogens at the population level.

## The Study

We screened 112 wastewater samples collected weekly over a 2-year period at a large regional treatment plant in Leuven for the presence of respiratory pathogens with an in-house–developed multiplex quantitative PCR respiratory panel (Appendix) (*4*). We investigated whether respiratory viruses found in wastewater corresponded to their detection in samples from patients with respiratory infections at the University Hospitals Leuven (UZL) (*5*). At UZL, patient samples were only tested with the respiratory panel in case of serious lower respiratory tract infection in immunocompromised or critically ill patients. Those clinical samples are therefore not entirely representative of locally circulating respiratory pathogens, especially when those pathogens cause mainly mild infections. When possible, we supplemented clinical sample data with epidemiologic data from sentinel laboratories across Belgium reported by Sciensano, but these were only available for a limited number of viruses (*6*). Nonpharmacologic interventions during the COVID-19 pandemic affected timing and levels of virus circulation.

**Figure 1:**
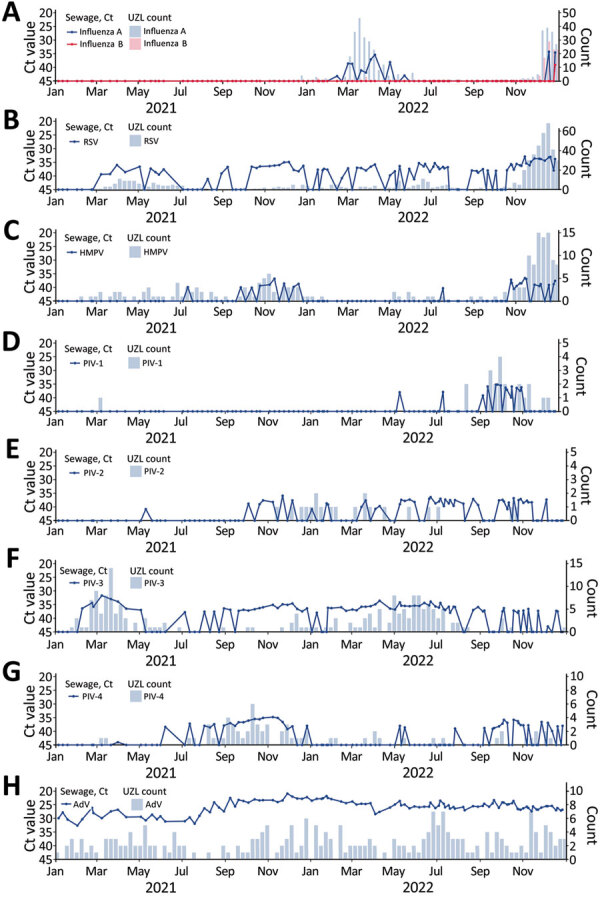
Respiratory viruses measured in wastewater versus positive clinical samples, Leuven, Belgium, January 2021–December 2022: A) influenza virus; B) RSV; C) HMPV; D) PIV-1; E) PIV-2; F) PIV-3; G) PIV-4; H) AdV. Graphs indicate evolution of viruses detected in wastewater by an in-house–developed multiplex quantitative PCR respiratory panel (line graphs; dots represent individual measurements) and by weekly counts of PCR-positive tests detected at UZL (bar graphs). Plots were generated using R version 4.1.1 (The R Foundation for Statistical Computing, https://www.r-project.org) and the ggplot2 package version 3.3.5 (https://ggplot2.tidyverse.org). A larger version of this figure is available at https://wwwnc.cdc.gov/EID/article/30/1/23-1011-F1.htm. AdV, adenovirus; Ct, cycle threshold; HMPV, human metapneumovirus; PIV, parainfluenzavirus; RSV, respiratory syncytial virus; UZL, University Hospitals Leuven.

Influenza A was repeatedly identified during mid-February–mid-May 2022 (Figure 1, panel A). This pattern aligned with positive clinical samples at UZL, which showed an influenza A peak during March–May 2022 and few cases outside that period. It also corresponded to Sciensano data, which indicated a mild 2020–21 influenza season and a late 2021–22 season (end of February to end of April) (*6*), caused almost exclusively by influenza A (*7*). The onset of the 2022–23 influenza epidemic, with cocirculation of influenza A and B, was reflected in positive wastewater samples as of mid-December.

The off-season peak of respiratory syncytial virus (RSV) in the spring of 2021, visible in clinical samples at UZL and in data reported by Sciensano, was reflected in positive wastewater samples during March–July 2021 (Figure 1, panel B). We detected RSV in almost all wastewater samples from mid-October 2021 until the end of July 2022. Data from Sciensano also showed a low continuous RSV presence in the 2021–22 season (*6*). After August 2022, RSV reappeared in wastewater; levels were elevated in November and December 2022. The number of positive clinical samples in UZL and sentinel laboratories remained low until the end of October 2022, followed by a strong RSV epidemic in November and December 2022.

During late September–December 2021, human metapneumovirus was almost continuously detectable in wastewater, which corresponded with high numbers of positive samples at UZL (Figure 1, panel C). Human metapneumovirus reappeared in wastewater in late October 2022, followed by an increase in positive samples at UZL in November and December.

Parainfluenzavirus (PIV) type 1 was predominantly found in wastewater samples during fall 2022, coinciding with a rise in positive cases at UZL. PIV-2 was sporadically detected in wastewater beginning in fall 2021, corresponding with low positive case numbers at UZL during November 2021–November 2022. PIV-3 was almost always detected in wastewater samples; a clear peak occurred during February–May 2021, in concordance with positive sample numbers at UZL. PIV-4 was detectable during August–December 2021 and September–December 2022, and occurred sporadically in between. The data also demonstrated an association with numbers of positive samples at UZL (Figure 1, panels D–G).

**Figure 2:**
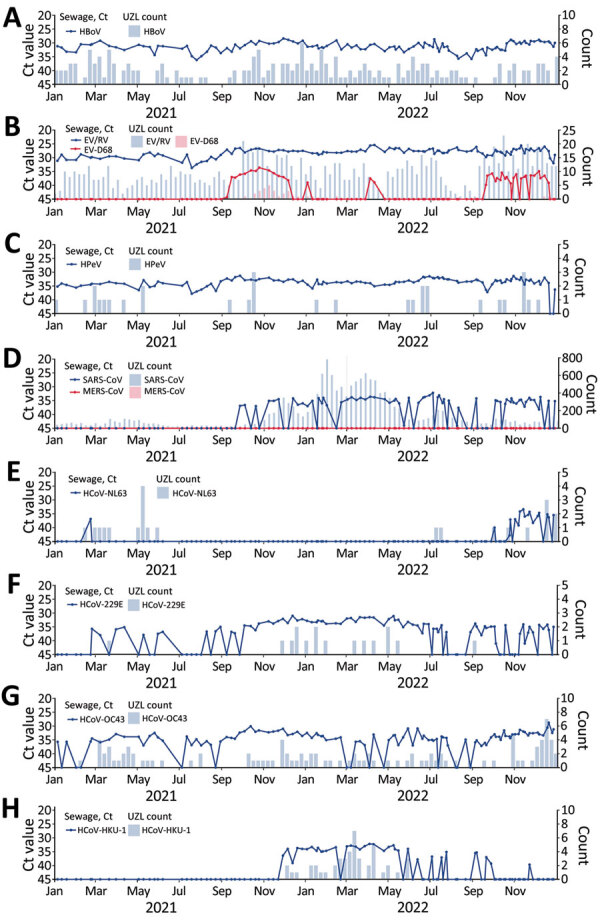
Respiratory viruses measured in wastewater versus number of positive clinical samples, Leuven, Belgium, January 2021–December 2022: A) HBoV; B) EV/RV and EV-D68; C) HPeV; D) SARS-CoV-1; SARS-CoV-2; and MERS-CoV; E) HCoV-NL63; F) HCoV-229E; G) HCoV-OC43; H) HCoV-HKU-1. Graphs indicate evolution of viruses detected in wastewater by an in-house–developed multiplex quantitative PCR respiratory panel (line graphs; dots represent individual measurements) and by weekly counts of PCR-positive tests detected at UZL (bar graphs). Plots were generated using R version 4.1.1 (The R Foundation for Statistical Computing, https://www.r-project.org) and the ggplot2 package version 3.3.5 (https://ggplot2.tidyverse.org). A larger version of this figure is available at https://wwwnc.cdc.gov/EID/article/30/1/23-1011-F2.htm. Ct, cycle threshold; EV, enterovirus; HBoV, bocavirus; HCoV, human coronavirus; HPeV, parechovirus; MERS-CoV, Middle East respiratory syndrome coronavirus; RV, rhinovirus; UZL, University Hospitals Leuven.

We detected adenovirus and human bocavirus (HBoV) consistently and in high concentrations in all wastewater samples (Figure 1, panel H; Figure 2, panel A). That finding is consistent with continuous adenovirus circulation in 2020–21 and 2021–22 reported by Sciensano and with our previous study on ARI samples, in which adenovirus infections were detected year-round (*8*). The continuous high-level detection of adenovirus and HBoV in wastewater does not align with the low numbers of positive samples found in ARI patients at UZL. Of the 4 HBoV genotypes, HBoV1 is mainly associated with respiratory symptoms in children with ARI and HBoV2 is linked to gastroenteritis (*9*,*10*). All HBoV genotypes are known to be present in stool and can frequently be detected in wastewater samples (*11*). Adenovirus infections can cause gastrointestinal symptoms, even when the primary site of involvement is the respiratory tract (*12*). The presence of HBoV and adenovirus in wastewater samples is likely linked to enteric rather than respiratory infections.

Enterovirus/rhinovirus were continuously detected in wastewater, but enterovirus D68 (EV-D68) was only present during early September–December 2021; the highest concentrations were detected in October 2021 (Figure 2, panel B). Those findings suggest a regional EV-D68 outbreak during fall 2021, in line with increasing EV-D68 infections in Europe in September 2021 (*13*). At UZL, 33 EV-D68–positive samples were detected during the study period, most during October 2021–January 2022. In mid-September 2022, EV-D68 reappeared in wastewater; only a small number of positive samples were reported at UZL. Detection of EV-D68 in wastewater preceded positive cases in the same region, indicating that wastewater surveillance can be used as a sensitive early warning signal for EV-D68 circulation.

Human parechovirus (HPeV) infections are common in children; illness can range from gastroenteritis and respiratory infections to neurologic disease, particularly in neonates (*14*). We detected HPeV consistently in almost all wastewater samples throughout the study but detected few positive clinical samples (Figure 2, panel C). HPeV’s presence in wastewater could be associated with enteric infections or paucisymptomatic respiratory infections with limited spillover to hospitals.

The SARS-CoV assay in the respiratory panel did not detect SARS-CoV-2 until late September 2021 (Figure 2, panel D). This assay targets a conserved region in the open reading frame 1ab polyprotein gene to enable detection of SARS-CoV-1 and SARS-CoV-2, resulting in a lower sensitivity. That lower sensitivity was observed in validation experiments on clinical samples but did not negatively affect accuracy in routine clinical practice (*4*). The assay is, however, not sensitive enough for environmental surveillance. Of the 4 endemic seasonal coronaviruses infecting humans, human coronavirus (HCoV) NL63 was primarily detected in wastewater during fall and winter of 2022, whereas HCoV-229E and HCoV-OC43 were present in most samples year-round. HCoV-HKU-1 was mainly detected between winter of 2021 and summer of 2022; all positive clinical samples were also reported during this period (Figure 2, panels E–H). Low numbers of HCoV positive clinical samples were detected in UZL, particularly for HCoV-NL63 and HCoV-229E, likely because of the mild nature of endemic coronavirus infections (typically not requiring hospitalization) rather than because of absence of circulation.

## Conclusions

By using an in-house respiratory panel to test a 2-year wastewater sample collection, we effectively detected the presence and seasonal variations of most tested respiratory viruses. These findings demonstrate wastewater sampling’s potential for population-level pathogen monitoring and early outbreak detection, addressing limitations associated with limited sentinel laboratory data. Our study underscores the role of wastewater-based epidemiology in supplementing clinical surveillance for respiratory viruses, enhances understanding of community virus circulation, and supports public health efforts.

Appendix Additional information for respiratory viruses in wastewater compared with clinical samples, Leuven, Belgium.
