# Supplementary material for: Respiratory Viruses in Wastewater Compared with Clinical Samples, Leuven, Belgium
Source: Emerg Infect Dis. 2024 Jan;30(1):141–5. doi: 10.3201/eid3001.231011 (PMC10756384; doi:10.3201/eid3001.231011)
Supplement: Appendix — Additional information for respiratory viruses in wastewater compared with clinical samples, Leuven, Belgium. [file 23-1011-Techapp-s1.pdf]

Article DOI: https://doi.org/10.3201/eid3001.231011

EID cannot ensure accessibility for supplementary materials supplied by authors. Readers who have difficulty accessing supplementary content should contact the authors for assistance.

Respiratory Viruses in Wastewater Compared with Clinical Samples, Leuven, Belgium

Appendix

Supplementary Data: Methods

Wastewater Sampling

Wastewater samples were collected on average weekly, starting January 5th 2021 up to December 28th 2022, from a large regional wastewater treatment plant (WWTP) in Leuven that treats municipal wastewater of ≈115000 inhabitants. Samples (500 mL) of 24-hour composite influent wastewater were collected through a time-proportional automated sampler, which collects 50 mL of wastewater every 10 minutes in a large container. The samples were stored in a refrigerator at 4°C before transport to the laboratory.

Viral concentration and TNA extraction

Virus concentration and filtration from the wastewater samples was performed as described previously (*1*). Total nucleic acids (TNA) were extracted from 500 µL concentrated and filtered sewage samples or DNA/RNA free water (negative controls) using the MagMAX Viral/Pathogen Nucleic Acid Isolation Kit on Kingfisher Flex-96 (ThermoFisher Scientific, Vilnius, Lithuania). TNA were eluted in 50 µL elution buffer. Extractions were done in duplicate to obtain sufficient volume of eluant for all the tests performed.

Respiratory panel (RP)

A qPCR RP for simultaneous detection of 22 respiratory viruses (influenza A, influenza B, RSV, HMPV, PIV-1 to −4, Adv, HBoV, RV/EV, EV-D68, HPeV, HCoV-NL63, −229E, -OC43, -HKU-1, -SARS and – MERS, CMV, HSV-1 and −2) and 7 bacteria/fungi (*Mycoplasma pneumoniae*, *Coxiella burnetii*, *Chlamydia pneumoniae*, *Chlamydia psittaci*, *Streptococcus pneumoniae*, *Legionella pneumophila* and *Pneumocystis jiroveci*) was

developed by the Department of Laboratory Medicine of UZL to allow quick diagnosis of respiratory pathogens in immunocompromised and/or critically ill patients with serious lower respiratory infection. This RP consisting of 12 real-time multiplex PCRs was performed on a QuantStudio 7 (ThermoFisher Scientific, Waltham, MA, USA) in 96 well plates. The end volume of each PCR reaction mix was 20 µL consisting of 5 µL of TNA, 5 µL of TaqMan Fast Virus 1-step Master Mix (Applied Biosystems, ThermoFisher Scientific, Vilnius, Lithuania) and 10 µL of primer/probe mix, with concentrations as published previously (*2*). The temperature profile used was 50°C for 10 minutes; 95°C for 20 seconds; 45 cycles composed of 95°C for 3 seconds and 60°C for 30 seconds.

Specificity of this lab-developed RP was validated at the UZL diagnostic laboratory in a clinical context using External Quality Control (EQC) samples, virus cultures and clinical respiratory samples.

Since HSV-1, HSV-2 and CMV are not typical respiratory pathogens, and they are not included in the UZL report on respiratory pathogens for comparison, we did not include these viruses in our analysis.

The bacteria and fungi of the RP were also not included in the analysis since the current wastewater treatment method, developed for optimal viral enrichment, is unsuited to confidently detect bacteria and fungi.

Detection of PMMoV with RT-qPCR

Human fecal indicator pepper mild mottle virus (PMMoV) was analyzed in the wastewater as an internal extraction control, and to check for extensive differences in human waste input and/or rainwater infiltration, as described previously (*1*,*3*).

Respiratory pathogens in clinical samples

Positive test results for respiratory pathogens in clinical samples (including oro- and nasopharyngeal swabs, bronchal or endotracheal aspirates and bronchoalveolar lavages) for the period during which wastewater samples were investigated were retrieved from UZL, which drains most patients from the wider region around the city of Leuven. In this hospital, the RP is performed on clinical samples of hospitalized patients for specific clinical indications. Data on the weekly number of PCR positive samples for respiratory pathogens, detected in UZL with the RP and additional multiplex molecular tests for influenza and RSV are published in a graphical format on the UZL Web site (*4*). Raw data were extracted from these graphs and were plotted over time against the wastewater Ct values.

Weekly numbers of samples testing positive for EV-D68 in the diagnostic laboratory of UZL were obtained from the 'National Reference Center (NRC) for Enterovirus, including polioviruses and parechoviruses', associated with UZL/KU Leuven.

Weekly numbers of samples testing positive for SARS-CoV-2 in the UZL diagnostic laboratory were obtained from the 'NRC for Respiratory pathogens - COVID-19', associated with UZL/KU Leuven. This number includes samples testing positive with the RP but also with other single – and multiplex SARS-CoV-2 assays, implemented in UZL.

References:

1. Rector A, Bloemen M, Thijssen M, Delang L, Raymenants J, Thibaut J, et al. Monitoring of SARS-CoV-2 concentration and circulation of variants of concern in wastewater of Leuven, Belgium. J Med Virol. 2023;95:e28587. PubMed https://doi.org/10.1002/jmv.28587

2. Raymenants J, Geenen C, Budts L, Thibaut J, Thijssen M, De Mulder H, et al. Indoor air surveillance and factors associated with respiratory pathogen detection in community settings in Belgium. Nat Commun. 2023;14:1332. PubMed https://doi.org/10.1038/s41467-023-36986-z

3. Kitajima M, Sassi HP, Torrey JR. Pepper mild mottle virus as a water quality indicator. npj Clean Water. 2018;1:19.

4. Laboratoriumgeneeskunde UZ. Leuven. Weekly detection results of respiratory pathogens at UZ Leuven [in Dutch] [cited 2023 Apr 4]. https://www.uzleuven.be/nl/laboratoriumgeneeskunde/wekelijkse-detectieresultaten-respiratoire-pathogenen
